# Incidence, mortality and survival in multiple myeloma compared to other hematopoietic neoplasms in Sweden up to year 2016

**DOI:** 10.1038/s41598-021-96804-8

**Published:** 2021-08-26

**Authors:** Kari Hemminki, Asta Försti, Markus Hansson

**Affiliations:** 1 Faculty of Medicine and Biomedical Center in Pilsen, Biomedical Center, Charles University in Prague, 30605 Pilsen, Czech Republic; 2 Division of Cancer Epidemiology, German Cancer Research Center (DKFZ), Im Neuenheimer Feld 580, 69120 Heidelberg, Germany; 3 Hopp Children’s Cancer Center (KiTZ), Heidelberg, Germany; 4 Division of Pediatric Neurooncology, German Cancer Research Center (DKFZ), German Cancer Consortium (DKTK), Heidelberg, Germany; 5 Sahlgrenska Academy, 413 25 Göteborg, Sweden; 6 Sahlgrenska University Hospital, 413 25 Göteborg, Sweden

**Keywords:** Cancer, Health care

## Abstract

Survival in multiple myeloma (MM) has developed favorably over the past decades for reasons that have been ascribed to new medications and treatment. However, development of survival over a long period and comparison to other hematopoietic neoplasms (HN) is less well known. Here we used Swedish cancer data from the Nordcan database, spanning a 50-year period from 1967 to 2016, and analyzed 1- and 5-year survival data. As a novel type of analysis we calculate the difference in survival between year 1 and 5 which indicates how well survival was maintained in the 4-year period following year 1 after diagnosis. The relative 1- and 5- year survival increased constantly; the 5-year survival graph for women was almost linear. The difference between 1- and 5-year survival revealed that the 5-year survival gain was entirely due to the improvement in 1-year survival, except for the last period. Survival improvement in all HNs exceeded that in MM. The linear 5-year survival increase for female MM patients suggests a contribution by many small improvements in the first year care rather than single major events. The future challenges are to push the gains past year 1 and to extend them to old patients.

## Introduction

Hematopoietic neoplasms (HNs) are a diverse group of cancers which differ in cellular origin, disease progression and clinical presentation. In the global ranking of incident case numbers among all cancers, leukemia, non-Hodgkin lymphoma (NHL) and multiple myeloma (MM) occupy places 10, 11 and 26; in ranking of fatal cases the placing is 10, 12 and 22, respectively^1^. Treatment in many HNs has improved over the years and the survival increase in Hodgkin lymphoma has been among the first success stories in cancer treatment^2^. Subsequently improvements have been seen in survival of many types of HNs^3–7^. Chemotherapy, based on alkylating agents such as cyclophosphamide and melphalan, was used for many HNs from the 1960s onwards. In the late 1980s autologous stem cell transplantation (ASCT) was introduced in hematology, and for MM it was used in combination with high-dose melphalan treatment^8–10^. Since then treatment of MM diverged from other HNs as described in “Methods section”^11–14^. Typical of treatment of MM and many other HNs has been combination of several types of drugs and ASCT, and consideration of many variables including age, performance status, comorbidities, and eligibility for ASCT (fit patients, earlier limited to patients < 65 years, but today < 70 years)^10^.

Sweden is among the high-incidence countries for MM together with Australia, North America and Western Europe^15^. Sweden has a special role in the history of MM as the monoclonal protein was first described by Jan Waldenström in 1961, and he was an expert in many other gammopathies (see^8^). In addition to his clinical studies in gammopathies, he initiated epidemiological follow-up on these diseases^16^. The Swedish health care system has been largely free of charge to the population at large. Another advantage of focusing on Sweden is its high level cancer registry which was among the first nationwide cancer registries in the world^17^. The combination of the early Swedish history on MM, tradition of disease epidemiology and open health care system stimulated us to describe nation-wide epidemiology of MM in comparison to other HNs as the previous studies by Swedish hematologists have focused on survival in MM only^10,11,18^. With analysis of the incidence/mortality/survival patterns we try to understand factors underlying improvements in survival in MM patients over a 50-year period in comparison to other HNs. We use the Nordcan database in the analysis, for which data were derived from the Swedish Cancer Registry.

## Results

The Nordcan database included 1.01 million male and 0.94 million female cancers for Sweden, excluding non-melanoma skin cancer, for years 1967 to 2016 (Table 1). The respective median diagnostic ages were 71 and 68 years. For HNs (including MM), the patient numbers were 91,444 and 73,922 and the median diagnostic ages were 68 and 66 years. In the same period, the database included 14,742 male and 12,145 female MM patients; these were 16.1% and 16.4% of all MN patients. MM patients included 4482 (30.4%) men who were diagnosed at age below 65 years, the age limit often applied for ASCT; 3069 (25.3%) women were diagnosed before that age. Additionally, 5326 (36.1% of all) men and 5167 (42.5%) women were diagnosed at age over 74 years that are often excluded from international statistics and clinical trials. The median diagnostic ages for MM in men and women were 71 and 73 years, respectively. In the first 10-year period the diagnostic ages were 69 years for men and 70 years for women; in the last 10-year period they were 71 and 73 years, respectively.

**Table 1:** Hematopoietic neoplasms and all cancers in Sweden 1967–2017.

Cancer	Men	Women
Hodgkin lymphoma	5911	4410
Non-Hodgkin lymphoma	29,097	23,721
Multiple myeloma	14,742	12,145
Leukemia	28,512	21,000
Myeloproliferative diseases	5945	6650
Myelodysplastic syndromes	3061	2369
Other malignant hematopoietic diseases	2638	2292
Malignant hematopoietic diseases	91,444	73,922
All cancers but non-melanoma skin cancer	1,007,049	941,127

**Figure 1:**
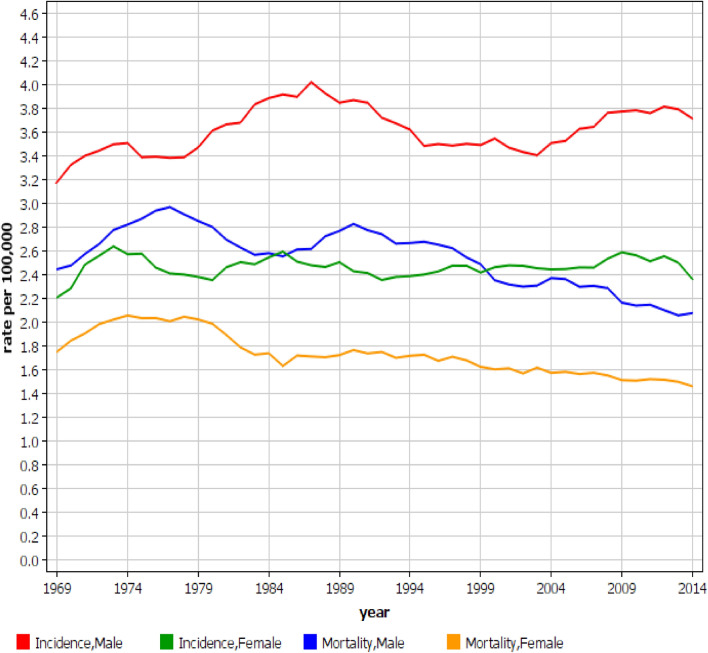
Incidence and mortality in multiple myeloma in Swedish men and women between 1967 and 2016. Note that because of the 3 year smoothing, the graphs do not show the full time span.

The incidence of MM in Swedish men peaked at 3.9/100,000 in around 1987 and modestly decreased but reached a new but lower peak at around 2010 (Fig. 1, note that because of smoothing the graphs do not exactly match these exact dates, nor the start and stop year). Mortality reached a maximum at 3.2/100,000 in 1976 with a subsequent decline to 2.5/100,000 by 2016. Among women an incidence maximum of 2.5/100,000 was reached in 1986 and remained at that level with some fluctuation up to 2016. The mortality peaked at 2.2/100,000 around 1977 and declined steadily to 1.7/100,000 by 2016 (Fig. 1).

**Figure 2:**
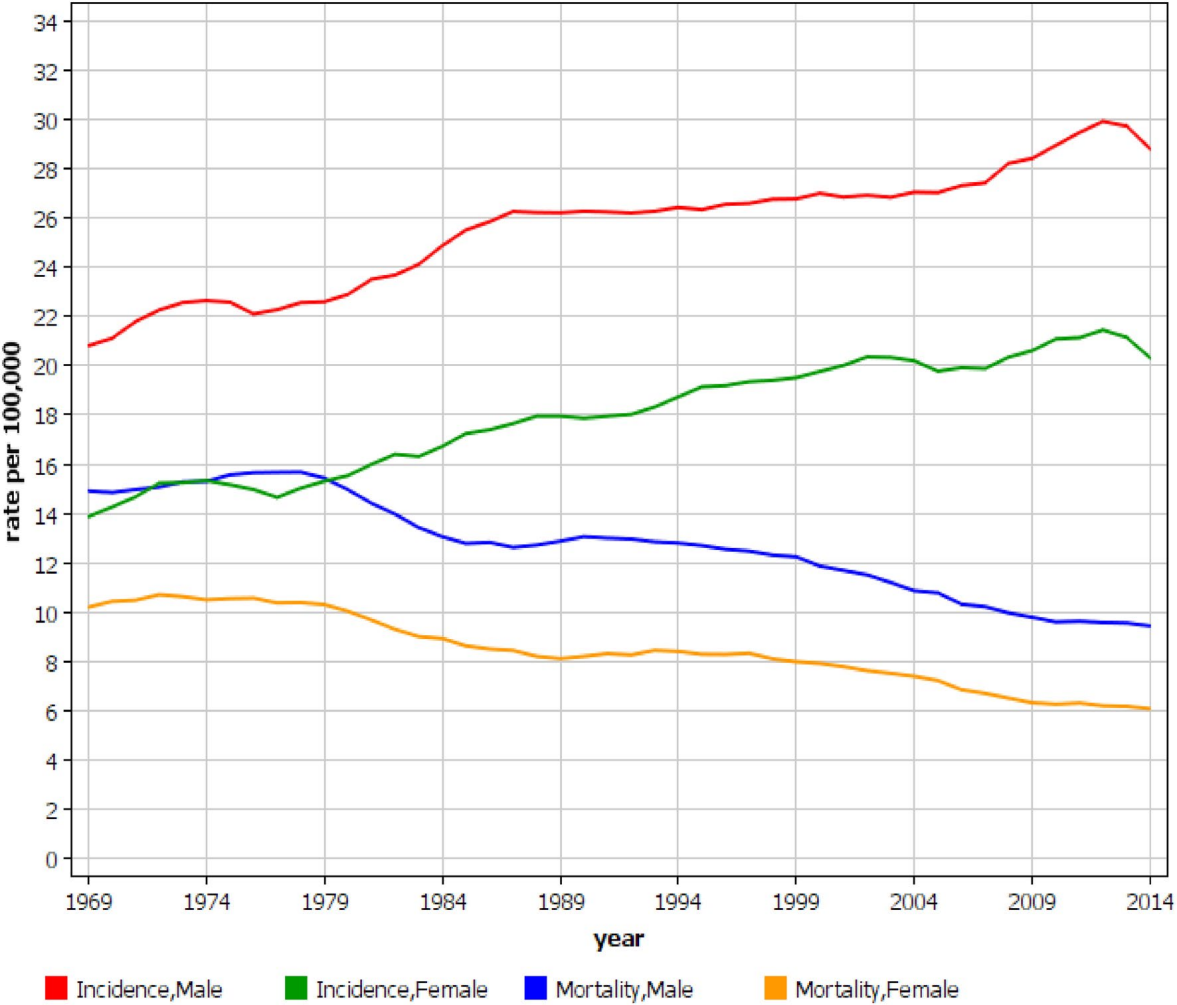
Incidence and mortality in hematopoietic neoplasms in Swedish men and women between 1967 and 2016. Note that because of the 3 year smoothing, the graphs do not show the full time span.

Data for HNs are shown in Fig. 2. A steady increase in incidence took place between years 1997 to 2012, male rates being higher than the female ones but with a parallel increase. In spite of the upward incidence, the mortality rates decreased, and the sex difference was narrowing in the course of time.

Relative 1-year and 5-year survival rates for MM are shown in Table 2. The male 1-year survival improved constantly from 54 (1967–1971) to 88% (2012–2016). The increases between the first two periods and between 2002–2006 and 2007–2012 were significant (i.e., 95%CIs were non-overlapping.). The male 5-year survival increased from 24% in 1967–1971 to 54% in 2012–2016; the increase between periods 2002–2006 and 2007–2012 was significant. The rightmost column shows the difference between 1- and 5-year survival in percent units (% units). Starting at 30% units in 1967–1971, it increased to 43% units in 1992–1996, and declined to 34% units in 2012–2016. For women, the 1-year survival increased from 64 to 88%, and the 5-survival increased from 25 to 53%. None of the changes between the periods were significant. The difference between 1-and 5-year survival started from 39% units and reached a peak of 44% units in 1997–2001 and declined again to 35% units. These changes are illustrated in Supplementary Fig. 1.

**Table 2:** Relative survival percent, 95% confidence intervals and survival difference between year 1 and 5 for multiple myeloma patients aged 0–89 years in Sweden 1 and 5 years after diagnosis.

Myeloma survival (%)					
Period		1-year	5-year		1–5 years%^a^
**Men**					
1967–1971	54	[51; 57]*	24	[21; 27]	30
1972–1976	65	[62; 68]	26	[23; 29]	39
1977–1981	67	[64; 70]	31	[28; 34]	36
1982–1986	72	[69; 74]	31	[28; 34]	41
1987–1991	74	[72; 77]	33	[31; 36]	41
1992–1996	77	[75; 79]	34	[32; 37]	43
1997–2001	78	[75; 80]	36	[34; 39]	42
2002–2006	80	[78; 82]*	41	[38; 43]*	39
2007–2011	86	[85; 88]	51	[48; 54]	35
2012–2016	88	[87; 90]	54	[52; 56]	34
**Women**					
1967–1971	64	[61; 67]	25	[22; 28]	39
1972–1976	67	[64; 70]	29	[26; 33]	38
1977–1981	71	[68; 74]	29	[26; 33]	42
1982–1986	72	[70; 75]	33	[30; 37]	39
1987–1991	77	[74; 79]	35	[32; 38]	42
1992–1996	80	[77; 82]	37	[34; 41]	43
1997–2001	83	[81; 85]	39	[37; 43]	44
2002–2006	83	[81; 85]	45	[42; 48]	38
2007–2011	85	[83; 87]	48	[46; 51]	37
2012–2016	88	[86; 90]	53	[50; 55]	35

^a^ 1–5 years% is the difference between survival percentages between year 1 and 5.

*Indicates that the 95%CIs between periodic survival percentages do not overlap (compared to the period below).

Survival data for all HNs are shown in Table 3. The male 1-year survival increased from 50 to 86% and the 5-years survival from 26 to 66%; because of the large case numbers most changes between the 1- and 5-year periods were significant. The male difference between 1-year and 5-year survival was initially 24% units, and reached a broad maximum at 28% between 1972 and 1991, finally declining to 20% units. For women, 1-year survival increased from 52 to 86% and for 5-year survival from 28 to 69%. The difference between 1-and 5-year survivals was initially 24% units, reaching a maximum of 28% units in 1977–1981, and thereafter declining to 17% units. These data are illustrated in Supplementary Fig. 2.

**Table 3:** Relative survival percent, 95% confidence intervals and survival difference between year 1 and 5 for hematopoietic malignancy patients aged 0–89 years in Sweden 1 and 5 years after diagnosis.

Hematopoietic neoplasm survival (%)					
Period		1-year	5-year		1–5 years%^a^
**Men**					
1967–1971	50	[49; 51]*	26	[24; 27] *	24
1972–1976	57	[56; 58]	29	[28; 31]	28
1977–1981	63	[62; 64]*	35	[34; 36]*	28
1982–1986	68	[67; 69]*	40	[39; 41]*	28
1987–1991	71	[70; 72]*	43	[42; 45]*	28
1992–1996	75	[74; 76]	48	[47; 50]*	27
1997–2001	77	[76; 78]*	52	[51; 53]*	25
2002–2006	81	[80; 82]*	59	[58; 60]*	22
2007–2011	84	[83; 85]	64	[63; 65]*	20
2012–2016	86	[85; 86]	66	[65; 67]	20
**Women**					
1967–1971	52	[50; 53]*	28	[27; 30]*	24
1972–1976	59	[58; 60]*	33	[32; 35]*	26
1977–1981	65	[64; 66]*	38	[36; 39]*	28
1982–1986	70	[69; 71]*	43	[42; 45]*	27
1987–1991	74	[73; 75]*	48	[46; 49]*	26
1992–1996	77	[76; 78]*	53	[51; 54]*	24
1997–2001	79	[79; 80]*	57	[55; 58]*	22
2002–2006	81	[81; 82]*	62	[61; 63]*	19
2007–2011	86	[85; 86]*	67	[66; 69]*	19
2012–2016	86	[86; 87]	69	[68; 70]	17

^a^ 1–5 years% is the difference between survival percentages of year 1 and 5.

*Indicates that the 95%CIs between periodic survival percentages do not overlap, compared to the period below.

**Figure 3:**
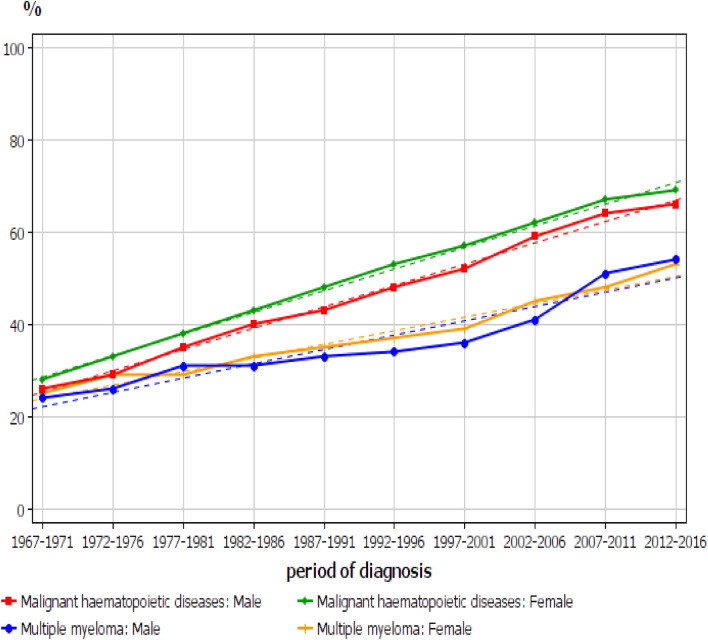
The 5-year relative survival percentage between all hematopoietic neoplasms and MM. The broken lines show the estimated trend.

The comparison of the 5-year relative survival percentage between all HNs and MM is shown in Fig. 3. The curves for all HNs are practically linear with a minor downturn towards the last period. The survival graphs for MM crossed between men and women. While the survival for women increased almost linearly, the male graph showed the relatively flat part from 1997–1981 to 1997–2001 and the steep increase thereafter to 2007–2011 and catching up with the female rate. The survival gap between all HNs and MM increased until about year 2000 and thereafter remained relatively constant. The 1-year survival graphs are shown in Supplementary Fig. 3. All the graphs were curvilinear with a steeper increase in the initial period and levelling off at around 1990 and thereafter a close to a linear increase. For MM, 1-year survival was initially somewhat higher than that for all HNs and for women the higher survival lasted until the last two periods.

In order to explain the survival gap between all HNs and MM we analyzed 5-year survival in the main HN subtypes (Supplementary Fig. 4). Male and female curves cluster together. Hodgkin lymphoma survival was most favorable but the shape of the curves for it differed from the others in having a steep early rise and slow levelling off while the other curves rose relatively linearly. NHL was second in survival ranking, followed by leukemia, then MM and finally myelodysplastic syndrome. Each of these main subtypes are heterogeneous and, for example for leukemia, chronic lymphocytic leukemia has had a very favorable development compared to some other types of leukemia (data not shown).

**Figure 4:**
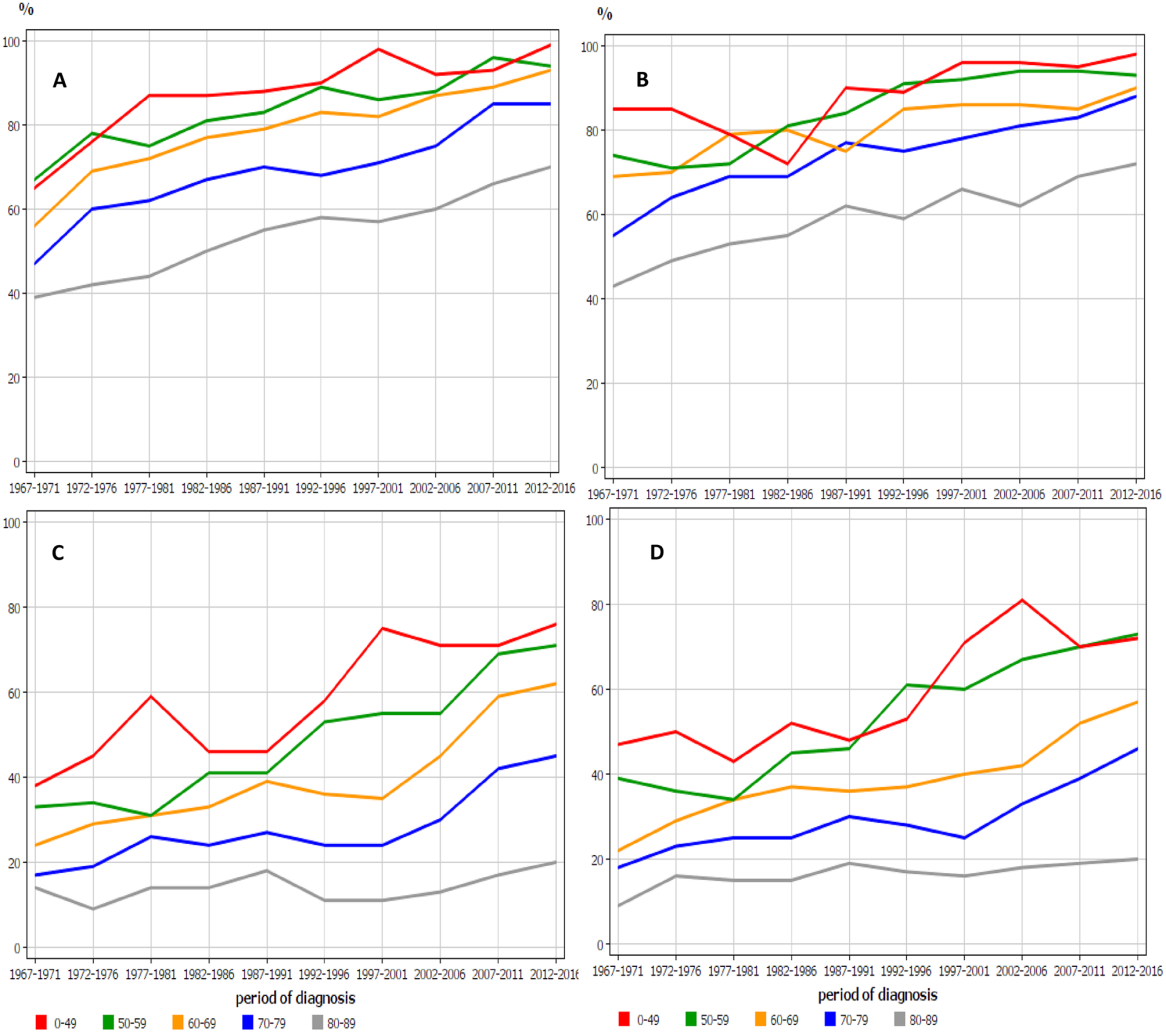
Age-specific 1-year relative survival for MM in men (**A**) and women (**B**) and age-specific 5-year relative survival in men (**C**) and women (**D**).

An age-and period-specific relative survival data for MM are shown in Fig. 4. In the early period, female 1- year relative survival (B) was better than male survival (A), particularly in the younger age groups but the differences disappeared towards the end of the follow-up. This can be confirmed in Table 2 where female survival (64%) significantly exceeded male survival (54%) in the first period but in the last period both had reached 88%. For 5-year survival (C and D) no male–female differences were evident. The concerns were the large age-group differences which for 5-year survival were widening in time and for the oldest age group hardly any survival gain was evident over the 50 year period (Fig. 4).

Default settings in Nordcan did not allow statistical evaluation of age-group specific survival data. We thus assessed mortality differences in age-groups for period 1967–2016 (Supplementary Table 1). Estimated annual percent change (EAPC) for MM mortality significantly declined for men and women at age 0–69 years. Mortality declined also in age group 70–70 years, and for women the declined was significantly less than in the younger age group. For the oldest men and women mortality modestly increased.

## Discussion

Progress in cancer control is measured by improvements in survival but in order to properly interpret this measure it is important to do it in the context of the other related epidemiological measures of incidence and mortality^19^. As the incidence in MM has been relatively stable and as the mortality has declined during the past 50 years in Sweden, the implication is that survival is improving which has been documented in several previous studies^11,20,21^. A recent study, with the title “Dramatically improved survival in multiple myeloma patients in the recent decade…”, described the follow-up from 1973 to the end of year 2013 and concluded that “…his progress is due to the revolutionary changes in the therapeutic arsenal and supportive care”^21^. Improved survival in MM has also been described for many other populations^7,9,22,23^. The overwhelming conclusion of these studies has been that therapeutic changes, particularly the novel agents, have contributed to the favorable development. Our overall results agree with the previous survival data but the present analysis with a follow-up from 1967 until 2016, separating male and female rates, and comparing to all HN, is able to provide some precision to the previous conclusions. Of note, the Swedish Cancer Registry data include 15–20% asymptomatic SMM (Supplementary Figure 5). These have been part in all Swedish MM related survival studies, except in the one collecting data from the Swedish Myeloma Registry, and showing better survival for SMM than for MM^10^. What the proportion of SMM has been before 2008 is not known.

For women the 5-year survival increased almost linearly over the whole follow-up period, which implies constant improvement in care without major events (Fig. 3). The male 5-year survival was initially at the level of the female survival but started to lag in the early 1980s and was followed by a catch-up starting in the late 1990s. The lag was mainly contributed by the weaker male improvement in the 1-year survival during that period (Supplementary Figure 3). The differences between 1-year and 5-year survival may also be telling in this regard (Table 2). For men, the difference between 1- and 5-year survival increased to 43% units in 1992–1996 and then declined to 34% units in 2012–2016. For women, the difference between 1-year and 5-year survival increased to years 1997–2001 and then declined below the level in 1967–1971. This suggests that constant gains were achieved in the 1-year care while the care between years 1 and 5 relatively worsened until the end of the 1990s and only thereafter improved, but the gains in survival between year 1 and year 5 were modest. This is also evident in the published survival data although it has attracted little attention. In the above Swedish study survival data were presented even for 3 months and 10 years^21^. The difference between the survival rates remained essentially constant over ca. 30 years indicating that survival gains in 10-year survival were contributed by improvements in 3-month survival.

The age-group specific analysis showed that the 5-year survival gains from year 1997 onwards benefitted patients aged from 50 to 79 years; for patients aged 50 to 59 years the positive development started already from 1977 onwards (Fig. 4). The male catch-up of the female 5-year survival starting in the late 1990s and coincided with the introduction of high-dose melphalan-ASCT. However, this cannot be the only reason because survival increased also among 70–79 year old men who rarely receive that treatment. The development was worrisome for oldest age groups as the survival gap to the youngest widened over time (Fig. 4). The implication is that the positive trend in 1-year survival for the oldest patients did not benefit 5-year survival as it did in the younger age groups. This sad conclusion was confirmed in the analysis of mortality trends. While mortality in MM declined in all other age groups, it modestly increased in those aged 80 + years. A recent Swedish study showed that over half of MM patients have comorbidities and these correlated with age and survival disadvantage^24^. Thus competing fatal causes are likely to be most prevalent among the old.

Survival in all HNs has been more favorable than that in MM, in spite of lower starting level in 1967–1971. This is particularly true for the small survival loss between years 1 and 5. Compared to MM, the decline in the difference between year 1 and 5 for HN started earlier and the last difference (20 and 17% units for men and women) was well below the first difference (24% units for both sexes).

Improvement in survival in MM has been reported by several authors. A previous study from Sweden, covering a period from 1973 to 2003 concluded that the likely causes to the positive development were high-dose melphalan with subsequent ASCT, thalidomide, and a continuous improvement in supportive care measures^11^. Kyle and Rajkumar also ascribed the improvements to novel therapies with thalidomide, bortezomib and lenalidomide^23^. Bergsagel came to a similar conclusion by applying the Surveillance, Epidemiology, and End Results (SEER) data which showed a steep increase in survival between 1999 and 2004, which he associated with the introduction of thalidomide in 1999 and of bortezomib in 2002^22^. The positive trend in the SEER population continued towards 2010, as did the German MM survival^7^. Turesson and coworker reviewed the literature and concluded that increase in relative survival is most likely related to the introduction of high-dose melphalan-ASCT, and later proteasome inhibitors and immunomodulatory drugs in younger patients but has been more modest in older patients, most of whom are not eligible for melphalan‐ASCT^9^.

Our results on MM show, firstly, that the main survival gain has been in the first year after diagnosis. Secondly, for women the steady improvement cannot be ascribed to any single major change in care but rather to multiple changes taking place over time. Thirdly, in men the rapid improvement in 5-year survival between in the late1990s was coincident with the introduction of melphalan‐ASCT but antedated a wide use of the novel agents. However, as the survival benefit was also among the 70 to 79 year old men, who rarely underwent ASCT therapy, it is likely that other factors contributed, and men were able to catch the beneficial conditions that promoted survival in women. Infections and renal failure are important causes of death in MM patients and their control probably contributed to the gains in early survival^25–27^. However, it is likely that other factors have helped improve survival over the 50-year period, such as diagnostic activity, facile start of treatment, control of comorbidities and overall patients care. Limitations of the study are that we have no individual data on such factors or treatment. The challenges in MM care are the old patients and patients at any age beyond year 1 after diagnosis including those with refractory disease and comorbidities.

## Methods

The data used originated from the Nordcan database which is a compilation of data from the Nordic cancer registries as described^28^. These registries are presented in detail by Pukkala and coworkers^17^. The database can now be accessed at International Agency for Cancer (IARC) website (https://nordcan.iarc.fr/en/database#bloc2). The analyses were conducted interchangeably at the IARC at the Nordcan sites (https://nordcan.iarc.fr/en/database#bloc2). The records for each patient include sex, dates of birth, cancer diagnosis and death, cancer diagnosis according to International Code of Diseases (ICD) version 10 and country and region of residence. National life tables were used for the calculation of incidence, mortality and survival figures. Data on the Swedish MM patients were extracted from Nordcan where the follow-up was extended until death, emigration or loss to follow-up or to the end of 2016. For age standardization the world standard population was used.

Survival analysis was conducted among all or age-group specific patient groups; only 3 patients were diagnosed below age 20 years. All survival data are ‘relative survival’ which is defined as the ratio of the observed survival in the group of patients compared to the survival expected in the general population, adjusted for sex, age and calendar time at the time of diagnosis. Survival data were available from 1967 onwards and the analysis was based on the cohort survival method for the first nine 5-year periods from 1964 to 2011, and a hybrid analysis combining period and cohort survival in the last period 2012–2016, as detailed^20,29^. The Swedish life tables were used to calculate the expected survival. For statistical assessment of survival data, 95% confidence intervals (CIs) were provided for each 5-year survival percentage. Statistical significance was called when 95%CIs for two survival figure did not overlap.

We calculated also a difference in survival percent between year 1 and year 5 as a measure on how well survival is maintained between years 1 and 5. A small difference indicates high survival between years 1 and 5 after diagnosis. This measure may be more concretely described by the complementary mortality, X = 100%-survival %. Thus if 1-year mortality X is small (say 10% with 90% survival), and 5-year mortality is also small (say 20% with 80% survival) then mortality is low and survival is favorable in the interval between 1 and 5 years.

As the default setting in Nordcan did not allow statistical evaluation of age-group specific survival data, we assessed mortality in MM in age-groups for period 1967 to 2016. Age-standardized (world) rates/100,000 and estimated annual percent change (EAPC) with 95%CI was presented.

In graphic presentation, smoothing of data in 3-year intervals was used to help control the annual variation. In some analyses, the youngest and oldest age groups were not included because of low case numbers.

Data from the Swedish Myeloma Registry (https://statistik.incanet.se/myelom/) was used to assess the frequency of asymptomatic MM (smoldering myeloma) among MM notifications (Supplementary Figure 1). Smoldering myeloma was estimated to account for 18.6% of all MM cases between 2008 and 2015^10^.

### Treatment of MM in Sweden

Chemotherapy using alkylating agents such as cyclophosphamide and melphalan in combination with steroids prednisone or dexamethasone, was used for many HNs from the 1960s onwards. In the late 1980s ASCT was introduced in hematology, and for MM it was used in combination with high-dose melphalan treatment^8–10^. National treatment principles for MM have been described by Kristinsson and coworkers and Blimark and coworkers^10,11,21^. Interferon alfa was introduced in the late 1970s, used either as a single agent or in combination with chemotherapy, in induction and in maintenance treatment. From the mid 1990s, high-dose melphalan and ASCT was recommended as up-front treatment for young MM patients (below 65 years) or up to 70 years for patients with good performance status; by 2008 80% of patients below age 65 years received this treatment^11^. In 2005, vincristine, adriamycin, and dexamethasone, or similar combinations, were recommended as induction treatment before high-dose melphalan-ASCT. For patients older than 65 years, melphalan and prednisone or cyclophosphamide and dexamethasone were up-front treatments until 2004. Around the turn of the millennium a set of novel agents with new mechanisms of action were introduced, including thalidomide, bortezomib and lenalidomide. In the guidelines from year 2010, bortezomib and thalidomide became part of standard induction therapy^10^. For patients not eligible for ASCT, thalidomide or bortezomib were added to the melphalan and prednisone scheme. By 2003 it was estimated that less than half of patients had received thalidomide while the use of bortezomib was about to start^11^. According to the data from 2008, only 31% of the MM patients had received thalidomide, bortezomib or lenalidomide but the proportion increasing to 68% by 2012^10^. Novel agents beyond the first generation of proteasome inhibitors (bortezomib) and immunomodulatory agents (thalidomide) have rapidly emerged, including, most recently, many immune based therapies^11–14^. According to the Swedish myeloma register, 77% of patients aged under 66 years at diagnosis and 5% of older patients received high-dose melphalan–ASCT as first-line treatment in the period 2008–2015^10^. In addition to the age and performance status, risk stratification into standard and high-risk MM guides treatment strategy^30^. In Sweden, diagnostics, treatment and follow-up of MM is concentrated to hospital-based hematology centers, and no patients are seen at private hospitals.

## Supplementary Information

Supplementary Information.

## Data Availability

A public database was used as the source of data. Please see “Methods” section.
