# Supplementary material for: Incidence, mortality and survival in multiple myeloma compared to other hematopoietic neoplasms in Sweden up to year 2016
Source: Sci Rep. 2021 Aug 26;11:17272. doi: 10.1038/s41598-021-96804-8 (PMC8390646; doi:10.1038/s41598-021-96804-8)
Supplement: Supplementary file 1 — Supplementary Information. [file 41598_2021_96804_MOESM1_ESM.pptx]

## Slide 1
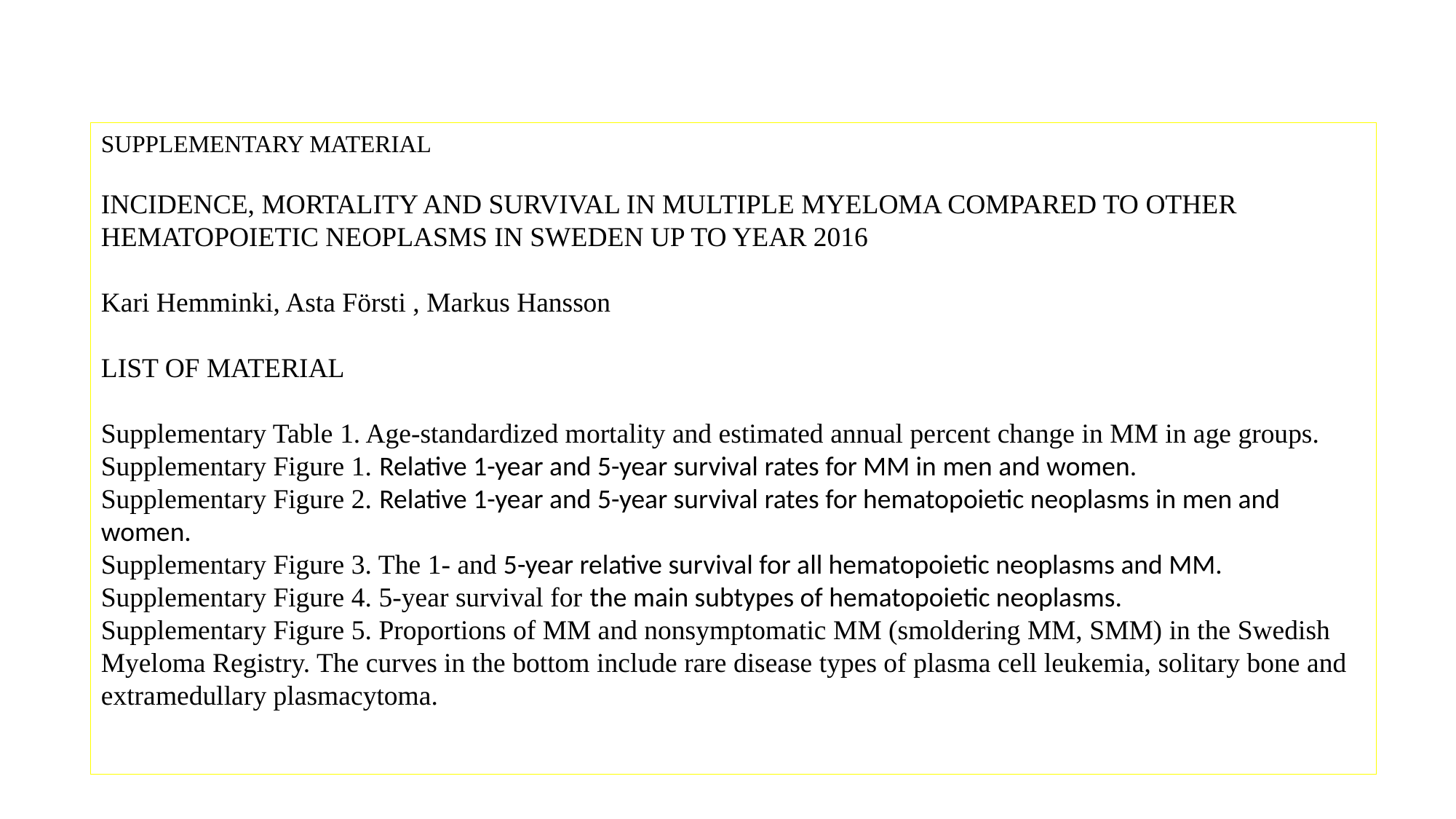

SUPPLEMENTARY MATERIAL
INCIDENCE, MORTALITY AND SURVIVAL IN MULTIPLE MYELOMA COMPARED TO OTHER HEMATOPOIETIC NEOPLASMS IN SWEDEN UP TO YEAR 2016
Kari Hemminki, Asta Försti , Markus Hansson
LIST OF MATERIAL
Supplementary Table 1. Age-standardized mortality and estimated annual percent change in MM in age groups.
Supplementary Figure 1. Relative 1-year and 5-year survival rates for MM in men and women.
Supplementary Figure 2. Relative 1-year and 5-year survival rates for hematopoietic neoplasms in men and women.
Supplementary Figure 3. The 1- and 5-year relative survival for all hematopoietic neoplasms and MM.
Supplementary Figure 4. 5-year survival for the main subtypes of hematopoietic neoplasms.
Supplementary Figure 5. Proportions of MM and nonsymptomatic MM (smoldering MM, SMM) in the Swedish Myeloma Registry. The curves in the bottom include rare disease types of plasma cell leukemia, solitary bone and extramedullary plasmacytoma.

## Slide 2
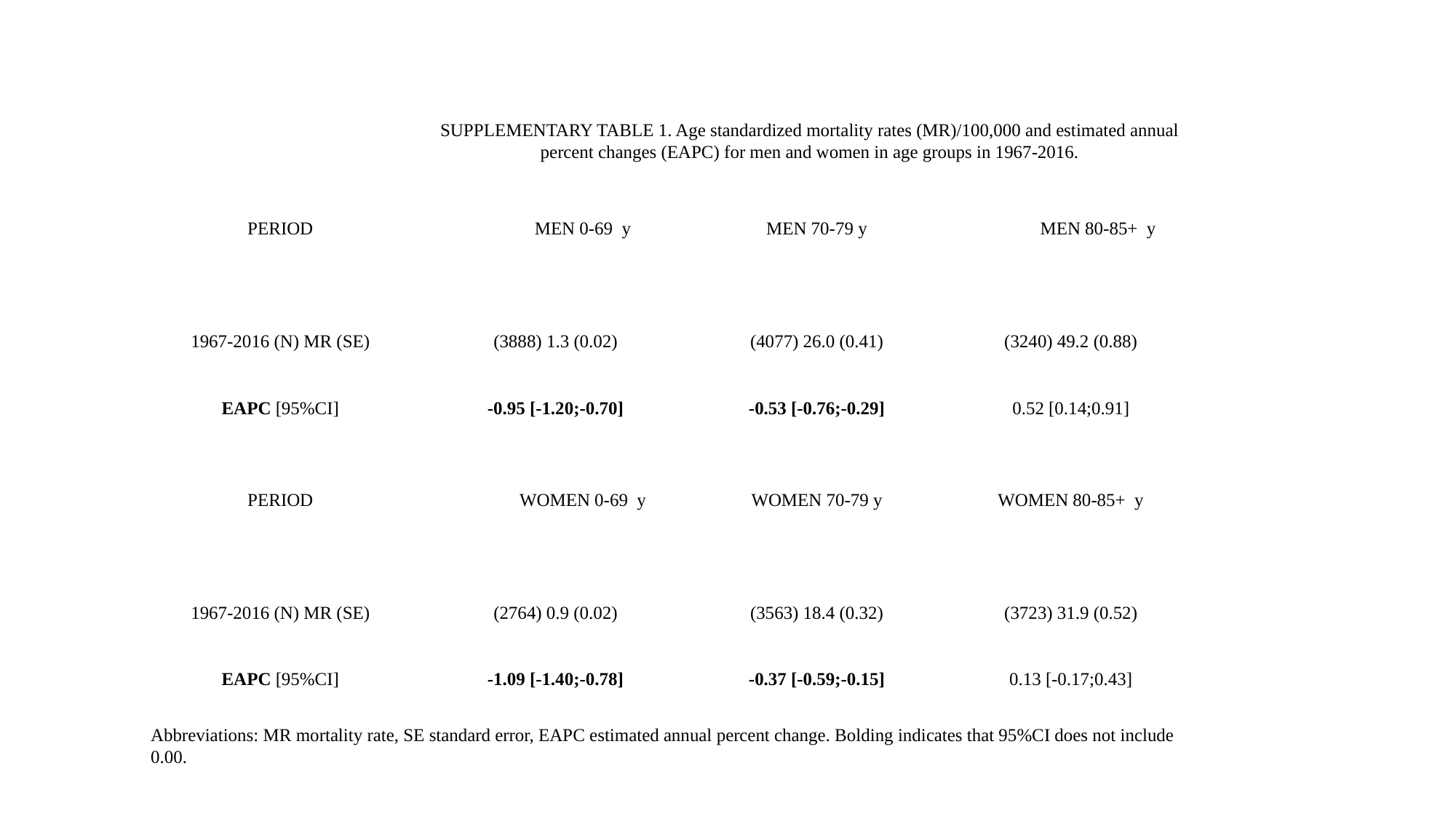

| | SUPPLEMENTARY TABLE 1. Age standardized mortality rates (MR)/100,000 and estimated annual percent changes (EAPC) for men and women in age groups in 1967-2016. | | |
| --- | --- | --- | --- |
| PERIOD | MEN 0-69 y | MEN 70-79 y | MEN 80-85+ y |
| | | | |
| 1967-2016 (N) MR (SE) | (3888) 1.3 (0.02) | (4077) 26.0 (0.41) | (3240) 49.2 (0.88) |
| EAPC [95%CI] | -0.95 [-1.20;-0.70] | -0.53 [-0.76;-0.29] | 0.52 [0.14;0.91] |
| | | | |
| PERIOD | WOMEN 0-69 y | WOMEN 70-79 y | WOMEN 80-85+ y |
| | | | |
| 1967-2016 (N) MR (SE) | (2764) 0.9 (0.02) | (3563) 18.4 (0.32) | (3723) 31.9 (0.52) |
| EAPC [95%CI] | -1.09 [-1.40;-0.78] | -0.37 [-0.59;-0.15] | 0.13 [-0.17;0.43] |
| Abbreviations: MR mortality rate, SE standard error, EAPC estimated annual percent change. Bolding indicates that 95%CI does not include 0.00. | | | |

## Slide 3
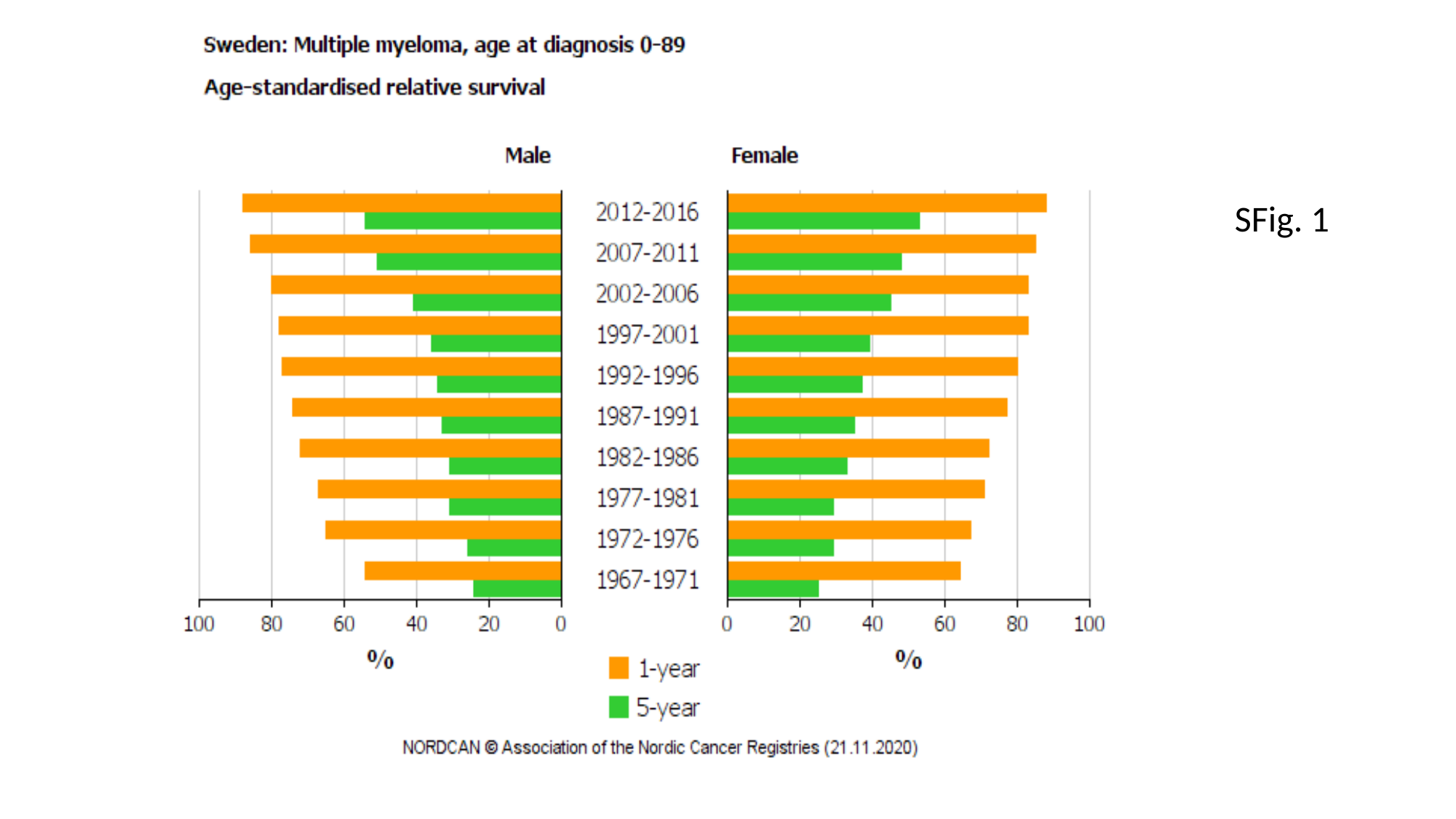

SFig. 1

## Slide 4
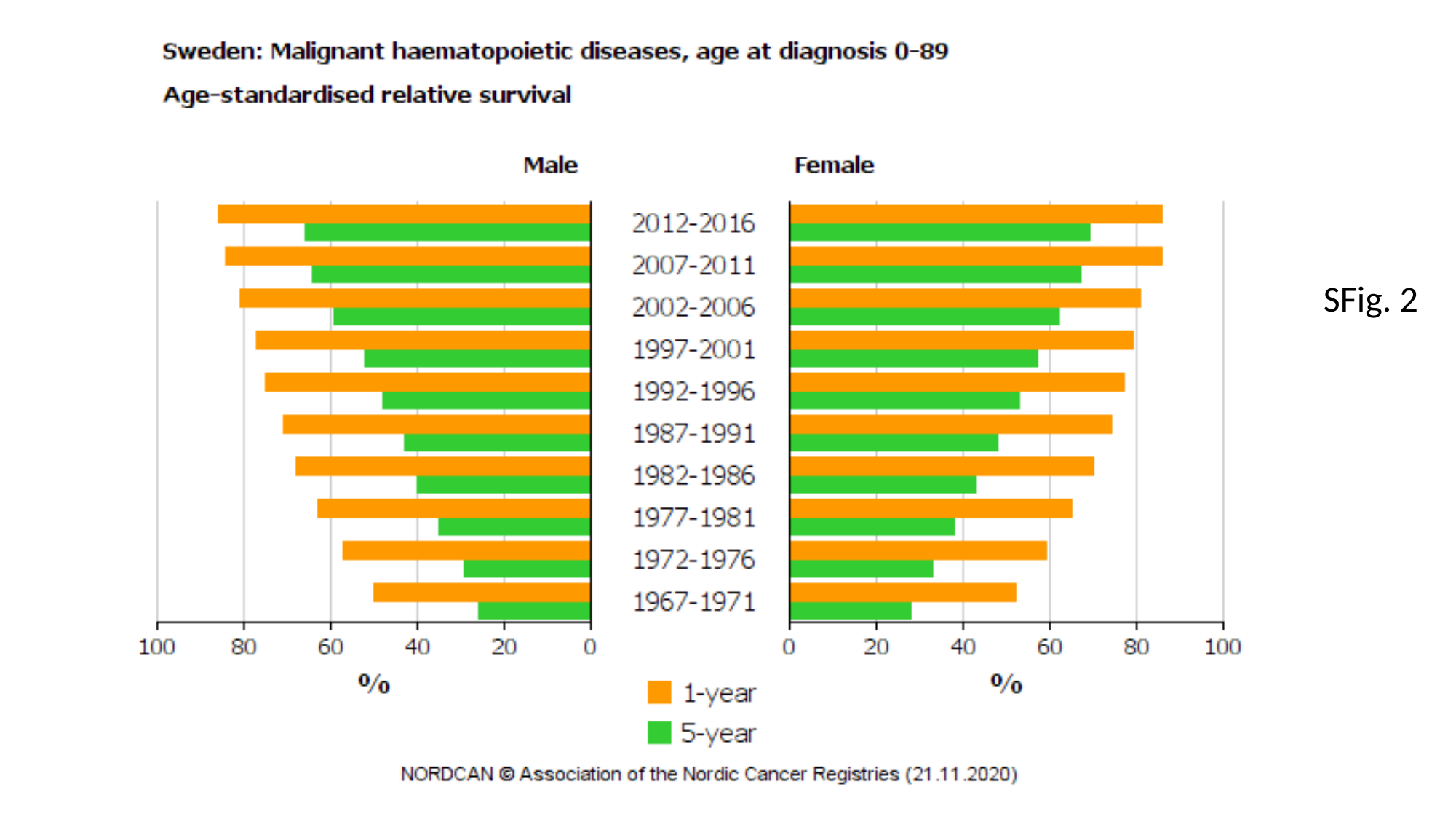

SFig. 2

## Slide 5
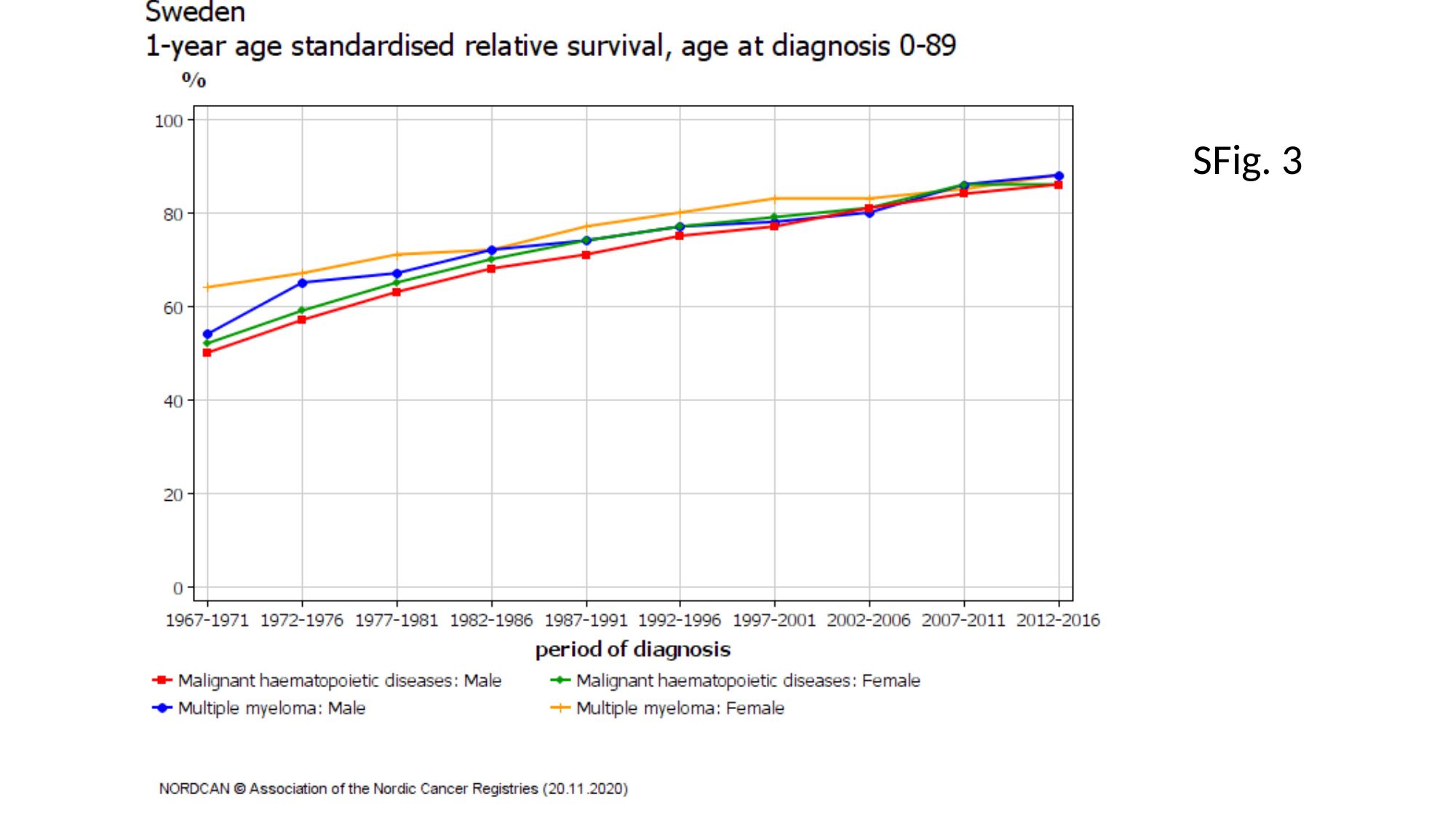

SFig. 3

## Slide 6
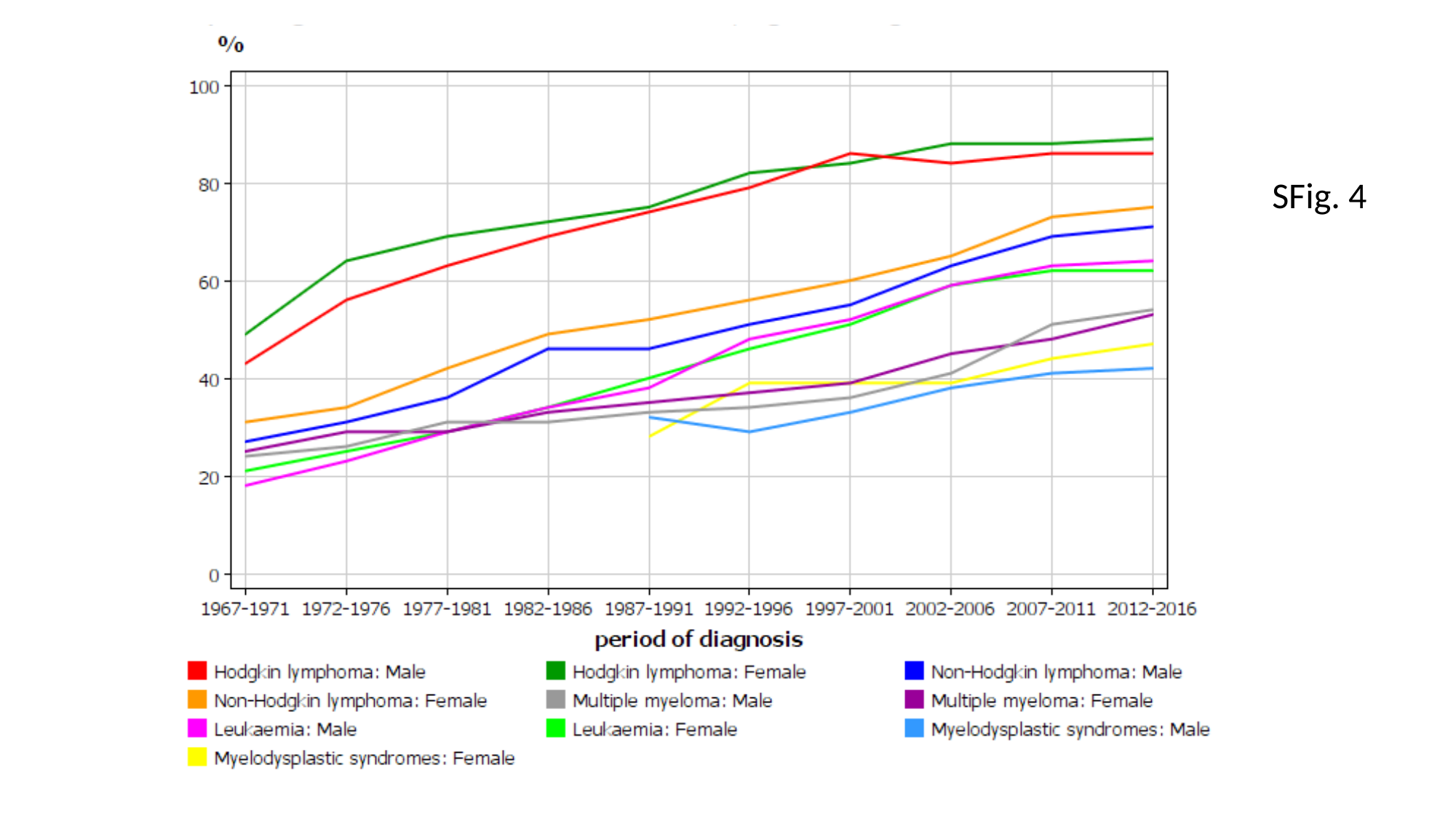

SFig. 4

## Slide 7
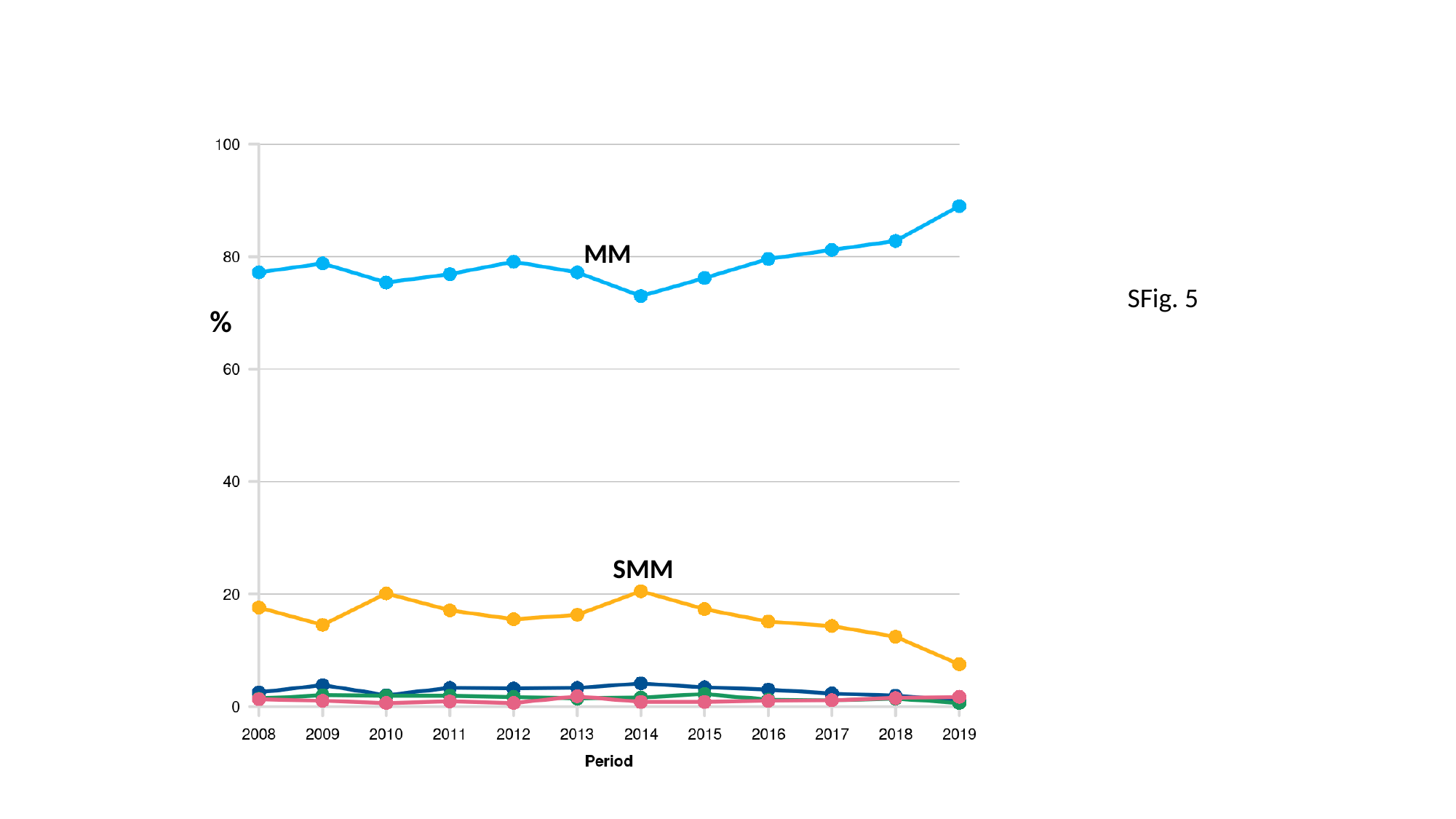

MM
SFig. 5
%
SMM
